# Pranoprofen Nanoparticles With *Poly*(L-*Lactide*)-b-*Poly*(*Ethylene Glycol*)-*b-Poly*(L-*Lactide*) as the Matrix Toward Improving Ocular Anti-inflammation

**DOI:** 10.3389/fbioe.2020.581621

**Published:** 2020-11-05

**Authors:** Yang Luo, Lu Yang, Peipei Feng, Haofeng Qiu, Xujin Wu, Shuwei Lu, Mi Zhou, Long Xu, Yabin Zhu

**Affiliations:** ^1^The Affiliated Hospital of Medical School, Ningbo University, Ningbo, China; ^2^School of Medicine, Ningbo University, Ningbo, China; ^3^School of Material Science and Chemical Engineering, Ningbo University, Ningbo, China

**Keywords:** PLEL, nanoparticles, pranoprofen, sustained release, ophthalmology

## Abstract

Nanotechnology using biodegradable polymer carriers with good biocompatibility and bioabsorbability has been studied and applied extensively in drug delivery systems and biomedical engineering. In this work, the triblocked oligomer poly(L-lactide)-*b*-poly(ethylene glycol)-*b*-poly(L-lactide) (PLEL) with the molecular weight of 2.08 KDa was first synthesized. Its chemistry was characterized by hydrogen nuclear magnetic resonance (^1^H-NMR) spectrum and Fourier transform infrared (FTIR) spectroscopy. Subsequently, the nanoparticles (NPs) of PLEL and pranoprofen (PF)-loaded PLEL were prepared with the average particle size of (151.7 ± 5.87) nm using the method of emulsion solvent evaporation. The formula and drug releasing profile were characterized by a transmission electron microscope (TEM), dynamic light scattering (DLS), and ultraviolet spectrophotometer (US). *In vitro* cytotoxicity assays and *in vivo* ophthalmic tests were performed to measure the safety and efficacy of the formulations. The results showed that PF NPs relieved the cytotoxicity of pure PF and eliminated ophthalmic irritation. The drug encapsulated in the nanoparticles displayed long-lasting release and good anti-inflammation efficiency in animal eyes. Therefore, we concluded that the present formula (PF NPs) could provide sustained drug release with good treatment effect on eye inflammation, and is promising for its use in ophthalmology in the future.

## Introduction

Ocular inflammation can cause general symptoms such as redness, swelling, pain, and even visual impairment in some severe cases. It mainly occurs through the action of several mediators such as histamine, bradykinin, interleukin, and prostaglandin (Sharma et al., 2016). Based on this pathophysiology, medicines including corticosteroids and non-steroidal anti-inflammatory drugs (NSAIDS) have been used for treatment or prevention of ocular inflammation (Soiberman et al., 2017; Wentz et al., 2019). At present, most clinical anti-inflammatory medicines are made as eye drops because this is the simplest and least invasive way to deliver drugs into the ocular tissues. However, eye drop administration means that less than 5% of the medicine will reach the necessary intraocular tissues, since reflex tear secretion will wash te drugs away or/and the drainage of the nasolacrimal duct will accelerate drug clearance (Hewitt et al., 2020).

Pranoprofen (PF), a non-steroidal anti-inflammatory drug, can relieve anterior eye inflammation and postoperative pain to alleviate clinical symptoms, such as redness, swelling, and pain, by inhibiting the release of prostaglandins (Abrego et al., 2015; Canadas et al., 2016; Rincón et al., 2018; Zhang et al., 2018). However, PF in eye drops has a low effective drug concentration and little bioavailability due to its poor water solubility (Abrego et al., 2014). Therefore, it is necessary to alter the administration pattern to improve the drug utilization.

Biodegradable polymers have been extensively studied before their potential use as drug delivery carriers, through which the drug can be released steadily under the degradation of the polymer to reduce administration frequency but maintain drug efficacy (Nagai et al., 2014; Zhan et al., 2019). The nanotechnology further polishes the drug delivery systems (Jumelle et al., 2020). Biodegradable synthetic polymers are particularly attractive to researchers because they exhibit good stability, safety, and tunable biodegradability which allow drug lifetimes to be extended to achieve a sustained release. The carriers reported to date include natural polymers such as chitosan, albumin, hyaluronic acid (HA), gelatin, and alginate, and synthetic polymers including polylactic-co-glycolic acid (PLGA), polylactic acid (PLA), polycaprolactone (PCL), and their copolymers. These macromolecules are also used as drug carriers in treatments of ocular diseases like inflammation, glaucoma, and cataracts (Lee et al., 2017; Gonzalez-Pizarro et al., 2018; Zhou et al., 2019).

Among these materials, poly (L-lactide) (PLLA) is attractive because it has good biocompatibility and bioabsorbability and thus plays an important role in the fields of tissue engineering, drug delivery systems, and surgical sutures (Tamai et al., 2000; Okamura et al., 2009, 2013). However, its application has been limited because of its hydrophobicity, low degradation rate, and acidity of the degraded products. Poly(ethylene glycol) (PEG), on the other hand, is a polymer with good hydrophilicity, non-toxicity, and excellent biocompatibility (Zhao et al., 2017; Guo et al., 2020). The combination of PLLA and PEG would be a good matrix as drug carriers due to their complementary properties of hydrophilicity and biocompatibility. The introduction of PEG into PLLA could accelerate the copolymer’s degradation and decrease the acidity of the degraded products. Moreover, the safety of both polymers has been certified by the US Food and Drug Administration (FDA).

In this work, the tri-blocked oligomer poly(L-lactide)-*b*-poly(ethylene glycol)-*b*-poly(L-lactide) (PLEL) was first synthesized. The chemistry was proven with hydrogen nuclear magnetic resonance (^1^H-NMR) spectrometer and Fourier transform infrared (FTIR) spectroscopy. The molecular weight of the oligomer was measured to be 20.8 KDa with gel permeation chromatography (GPC). Subsequently, the PLEL nanoparticles (NPs) were prepared using the emulsion solvent evaporation method. The drug pranoprofen (PF) was further encapsulated into these nanoparticles to obtain pranoprofen-loaded nanoparticles (PF NPs). They were characterized by Transmission Electron Microscope (TEM) and Dynamic Light Scattering (DLS) equipment. *In vitro* cytotoxicity assays and *in vivo* ophthalmic tests were performed to ensure the safety and efficacy of this formulation.

## Materials and Methods

### Materials

PF was obtained from Shanghai Macklin Biochemical Technology Co., Ltd. (Shanghai, China). L-lactide (LLA) and Poly (vinyl alcohol) (PVA) were purchased from Sigma-Aldrich Shanghai Trading Co., Ltd. (Shanghai, China). Dichloromethane (AR) and polyethylene glycol (PEG, Mn = 2,000 Da) were purchased from Sinopharm Chemical Reagent Co., Ltd. (Shanghai, China). B4G12 human corneal endothelial cell line (HCEC-B4G12) was obtained from BeNa Culture Collection (Suzhou, China) and BV2 microglia cells were obtained from Shanghai Institutes for Biological Sciences (Shanghai, China). Fetal bovine serum (FBS) was obtained from Hyclone (Beijing, China) and the other reagents for cell culture were obtained from Gibco (Shanghai, China). The purified water used in all experiments was obtained from a MilliQ System (home supplied). All other chemicals and reagents used in the study were of an analytical grade.

New Zealand rabbits were supplied by the Animal Experiment Center of Ningbo University. They were housed at room temperature (24–25°C) with free access to food and water. All animal experiments were conducted according to the statement for the Use of Animals in Ophthalmic and Vision Research established by the Association for Research in Vision and Ophthalmology. All animal experiments were approved by the Animal Ethics Committee of Ningbo University.

### Preparation of PLEL

**FIGURE 1:**
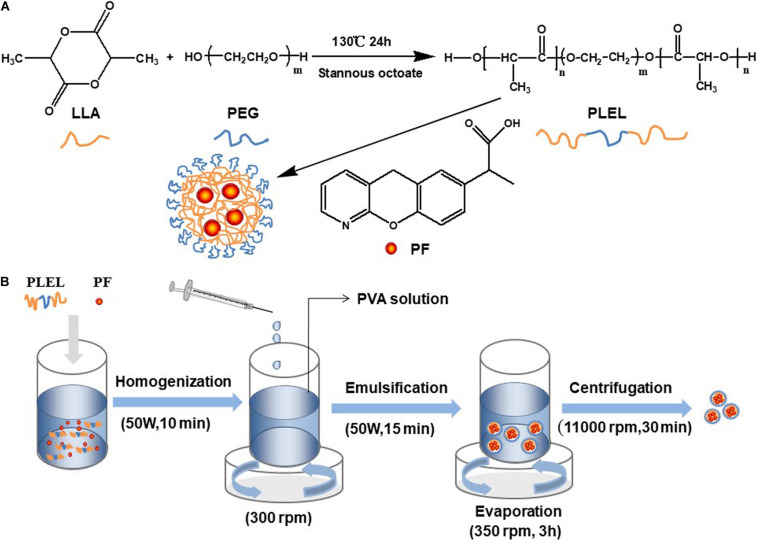
Schematic diagram. **(A)** PLEL polymerization. **(B)** PF NPs preparation.

Dry LLA and PEG with a mass ratio of 6:1 were mixed in a three-necked flask. The catalyst, stannous caprylate (0.1%, w/w), was added into the flask. The reaction took place at 130°C for 24 h. The triblock copolymers PLEL was thus obtained. Dichloromethane was used to dissolve the product after the reaction, and ethyl alcohol was used to precipitate the product. PLEL was collected by drying the sediment using a drying oven (DHG-9123A, Ningbo Jiangnan Instrument Factory, China). The reaction is schematically displayed in Figure 1A.

### Characterization of PLEL

The PLEL chemistry was measured on a Fourier transform infrared (FTIR) spectroscopy (TENSOR-27, Bruker Corporation, Germany) and hydrogen nuclear magnetic resonance (^1^H-NMR) spectrometer (Awance III-500, Bruker Corporation, Germany). The FTIR measurement was performed at a resolution of 4 cm^–1^ at a frequency interval of 400–4,000 cm^–1^. The ^1^H-NMR spectrometer was performed at a frequency of 500 MHz with deuterated chloroform (CDCL_3_) as the solvent. The molecular weight and molecular weight distribution of PLEL was measured by gel permeation chromatography (GPC) with tetrahydrofuran (THF) as the solvent.

### Preparation of Nanoparticles of PF/PLEL

PF was encapsulated into PLEL using emulsion solvent evaporation technology (Wang et al., 2018). In brief, 5 mg PF was added into 2 mL dichloromethane with 20 mg PLEL polymer. The solute was dispersed in an ice bath with ultrasonic equipment (ISO9001, Ningbo Power Ultrasonic Equipment, China). This dispersed solution was then added slowly into PVA solution (20 mL, 1 wt% in water) under stirring (300 rpm). This mixture was emulsified with ultrasonic equipment (50 W, 15 min) in an ice bath. Then, the emulsion was stirred (350 rpm) at room temperature for 3 h to allow the evaporation of organic solvent. The PF-loaded NPs (PF NPs) were thus obtained. These PF NPs were subsequently washed three times with purified water, each centrifuged at 11,000 rpm for 30 min on High-speed centrifuge (5840 R, Eppendorf Corporation, Germany), and freeze-dried for 48 h in a freeze-dryer (Freeze zone 2.5, Labconco Corporation, United States). The process of PF NPs’ preparation is schematically diagramed in Figure 1B. Preparation of nanoparticles of PLEL (PLEL NPs) was the same as above, but PF was not involved.

### Characterization of PF NPs

Surface morphology and particle size of PF NPs were observed under a transmission electron microscopy (TEM, JEOL-2100F, Japan) with 1% (w/v) phosphotungstic acid for particle staining. A dynamic Light Scattering (DLS) instrument (Autosizer 4700, Malvern, Britain) was used to analyze the particle size and distribution of PF NPs.

The critical aggregation concentration (CAC) of PLEL was measured on a Fluorescence spectrophotometer (RF-5301PC, Shimadzu, Japan). In brief, pyrene was dissolved in acetone at a concentration of 5.93 × 10^–7^ g.L^–1^. Pyrene/acetone solution (200 μL) was placed in a vial and acetone was allowed to volatilize at 50°C for 2 h. Polymer nanoparticles solution (5 mL) with different concentrations (from 5 × 10^–1^ to 5 × 10^–5^ g.L^–1^) were added into the vials, bathed at 65°C for 3 h, and placed in the dark for 24 h. The solution was measured on a Fluorescence spectrophotometer with an emission wavelength of 393 nm.

The entrapment efficiency (EE,%) and loading efficiency (LE,%) of PF NPs was determined as the equations:

(1) $$\begin{array}{c} \left(\mathrm{EE}\right)\left(\%\right)=\frac{\text{(total employed PF}-\text{PF in supernatant (mg))}}{\text{total employed PF (mg)}}\\ \;\times 100\% \end{array}$$

(2) $$\begin{array}{c} \left(\mathrm{LE}\right)\left(\%\right)=\frac{\text{(total employed PF}-\text{PF in supernatant (mg))}}{\text{nanoparticles mass (mg)}}\\ \;\times 100\% \end{array}$$

Where the standard curve of PF concentration (0.0–125.0 mg/L) as a function of the absorbance at the wavelength of 246 nm was setup. The absorbance was measured on an Ultraviolet spectrophotometer (U3010, Hitachi, Japan). It showed as:

(3) $$y=0.0260x+0.0559\left(\mathrm{regression}\mathrm{coefficient}r^{2}=0.99997\right)$$

### *In vitro* Drug Release

The release characters of PF from the nanoparticles were measured with the dialysis bag diffusion method (Chen et al., 2013). PF NPs (containing 5 mg PF) were resuspended in phosphate buffer solution (PBS, pH = 7.4) and placed into a dialysis bag (*M*w = 100–500 Da, Shanghai Yuanye Bio-Technology Co., Ltd, Shanghai, China). The bag was hermetically sealed and immersed into 50 mL PBS with magnetic stirring at 300 rpm at 37°C. At predetermined time intervals, 800 μL of the released solution was removed while an equal amount of fresh PBS was added to the solution. The removed solution was filtered through a 0.22 μm filter and analyzed for PF amounts by Ultraviolet spectrophotometer (U3010, Hitachi, Japan). The cumulative release of PF (%) was calculated as the equation:

(4) $$\begin{array}{c} \mathrm{Cumulative}\mathrm{release}\mathrm{of}\mathrm{PF}\left(\%\right)\\ \;=\frac{\text{Amount of PF released (mg)}}{\text{Amount of PF in nanoparticles (mg)}}\times 100\% \end{array}$$

## *In vitro* Cytotoxicity Assessment

Both HCEC-B4G12 cells and BV2 microglia cells were used to assess the cytotoxicity of materials by Cell Counting Kit-8 (CCK8) assay. HCEC-B4G12 cells were cultured in High Glucose-Dulbecco’s Modified Eagle Medium (H-DMEM) while BV2 microglia cells in DMEM, Nutrient Mixture F-12 (DMEM/F-12, 1:1). Both mediums were supplemented with fetal bovine serum (FBS) at the concentration of 10%. All cultures were conducted in an incubator (HF90, Heal Force, China) containing 5% CO_2_ at 37°C.

Cells of HCEC-B4G12 and BV2 were seeded, respectively, on the wells of 96-well culture plates at a density of 1.0 × 10^4^ cells per well and incubated at 37°C in a wet atmosphere containing 5% CO_2_ for 24 h. In order to test the cytocompatibility of materials, PLEL NPs in concentrations from 0 to 1,000 μg/mL were added into cells and cultured for more than 72 h.

Both cells of HCEC-B4G12 and BV2 were treated with PF NPs suspension and PF solution for 24–72 h, respectively, to detect toxicity. The final concentrations of PF NPs or PF were 0.04–0.8 mM in the culture. After incubation, the medium was removed and replaced with 110 μL fresh culture medium containing 10 μL CCK8 reagents and incubated in a dark environment for more 4 h at 37°C. Finally, 80 μL CCK8-containing medium for each well was pipetted and transferred into a new 96-well plate. The absorbance at the wavelength of 450 nm was measured on a microplate reader (Dragon Wellscan MK-3, Labsystems, Finland). The cell viability was calculated as follows:

(5) $$\mathrm{Cell}\mathrm{Viability}\left(\%\right)=\frac{\text{Absorbance of treated cell}}{\text{Absorbance of untreated cells}}\times 100\%$$

## Ophthalmic Irritation Assay

### Single-Dose Irritation Test

New Zealand rabbits (2.5–3.0 kg) were used as the testing animals. They were divided into four groups (*n* = 5). The right eye of each rabbit was treated while the left was left alone. Each group was given 50 μL of physiological saline (PS), PLEL NPs, PF, and PF NPs, respectively, with concentrations of 1 mg/mL. The ocular surface was evaluated with the Draize Eye Test at 1, 4, 8, and 24 h post-operation (Lai et al., 2019).

### Long-Term Irritation Test

Rabbits were treated with PLEL NPs, PF, or PF NPs (50 μL for each eye), respectively, three times daily for 7 days. All eyes were monitored under a slit lamp (SLM1-2ER, Kang Huaruiming, China). Sodium fluorescein was used to stain the eyes for assessment of the damage seriousness of the cornea. After treatment for 7 days, the eyes were collected and sectioned for ∼6 μm by freezing microtome (Cryostar NX50, Thermo, Shanghai, China). The sections were then stained by haematoxylin & eosin (HE) dyes, dehydrated gradientedly by ethanol aqueous solution (70, 80, 90, and 100%, each for 2 min), and cleared with xylene for 10 min. The sections were observed under a light microscope (CKX41SF, Olympus, Japan).

### *In vivo* Anti-inflammatory Assessment

The animal model with infected eyes was set up by an injection of LPS/PS solution (0.2 mg/ml) into the anterior chamber (Guo et al., 2019). The incision was made in the cornea margin of rabbits to allow the aqueous humor to flow naturally. LPS solution (0.12 mL for each eye) was injected into the anterior chamber of the animal eyes. They were divided into three groups with three animals per group (*n* = 3). The right eye of each rabbit was treated with PS, PF, or PF NPs (50 μL, 1 mg/mL), respectively, twice daily for 4 days. The left eye was not treated and was used as the control.

All eyes were monitored under a slit lamp for the whole experimental interval. The inflammation severity was evaluated with Scores of inflammation severity of ocular tissues, which refers to the Draize Eye Test method, and changed accordingly (Draize et al., 1944). The rules of the detail scores are shown in Table 1. The tissues of the cornea and iris were collected and stained with HE dyes for histological assessment after treatment for 4 days.

**TABLE 1:** Scores of inflammation severity of ocular tissues (Draize et al., 1944).

Score	Cornea	Anterior chamber	Iris		Conjunctival	
0	No haze	No exudates	No swelling or congestion	Normal pupillary reflex	No swelling or congestion	No tears or secretions
1	Mild haze	Mild exudates	Mild swelling or congestion	Sluggish pupillary reflex	Mild swelling or congestion	Mild tears or secretions
2	Moderate haze	Moderate exudates	Moderate swelling or congestion	–	Moderate swelling or congestion	Tears or secretions moisten the eyelids and lashes
3	Severe haze	Severe exudates	Severe swelling or congestion	–	Severe swelling or congestion	Tears or secretions moisten the entire eye area
4	Totally hazy cornea	Totally covered by exudates	–	–	–	–

### Statistical Analysis

All experiments were repeated at least three times. The data were presented as the mean ± standard deviation (SD) from at least three independent experiments. Statistical analyses were performed using SPSS 18.0 software via one-way analysis of variance (ANOVA) or *t-*test (*n* ≥ 3). A value of *p* < 0.05 indicated statistical significance.

## Results and Discussion

### Characterization of PLEL Copolymer

**FIGURE 2:**
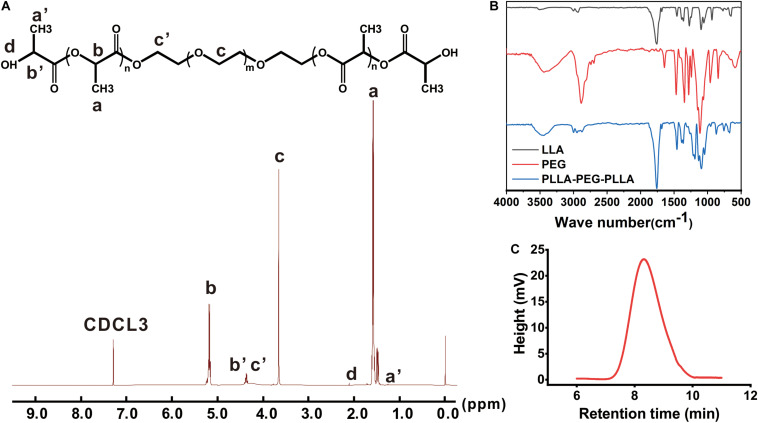
The characterization of PLEL. **(A)** ^1^H-NMR spectrum. **(B)** FTIR spectroscopy. **(C)** Molecular weight distribution.

The chemistry of the PLEL copolymer was tested by ^1^H-NMR spectrometer. As shown in Figure 2A, the resonance peaks corresponded to the characteristic bonds of the PLEL molecule, including 1.58 and 5.16 ppm (-CH_3_ and -CH in PLLA), 2.11 ppm (-OH at the end), and 3.65 ppm (-CH_2_ in PEG), suggesting that PLEL was successfully synthesized.

The molecular structure of PLEL was also measured by FTIR spectroscopy. The results showed that both black and red curves in Figure 2B characterized molecules of LLA and PEG, including the typical 1,759 cm^–1^ (C = O stretch) in LLA and 3,450 cm^–*l*^ (-OH stretch) in PEG. After the reaction, the peak at 3,450 cm^–*l*^ was weakened but the 1,750 cm^–1^ peak strengthened (Figure 2B, blue curve), which indicated the formation of an ester group that came from the ring opening reaction between LLA and PEG. Therefore, we concluded that PLLA-PEG-PLLA copolymer (abbreviated as PLEL) was successfully synthesized in our reaction protocol.

The molecular weight of PLEL copolymer was 20.8 KDa (Figure 2C). The relatively narrow GPC profiles without shoulder peaks indicate that the products are basically pure without obvious monomers.

### Characterization of PF NPs

**FIGURE 3:**
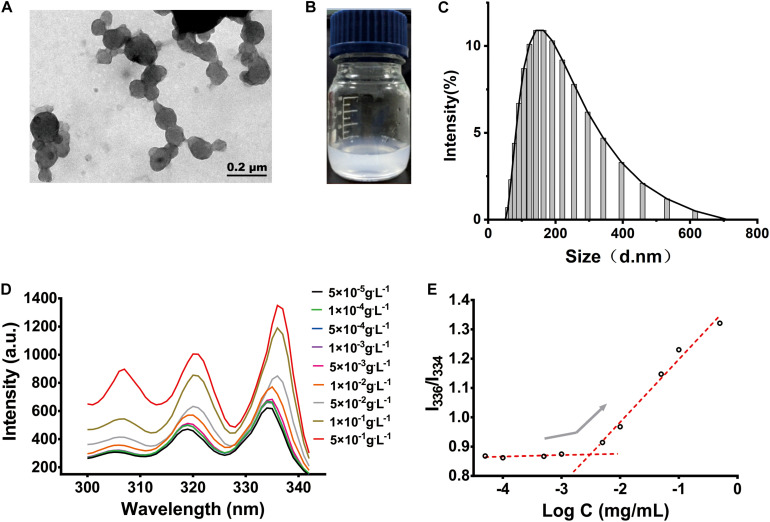
The characterization of PF NPs. **(A)** TEM image. **(B)** Eye size of PF NPs (0.2 mg/mL) dispersed in water. **(C)** Size distribution. **(D)** Fluorescence excitation spectrum. **(E)** CAC determinations of PLEL.

The morphology of the PF NPs was observed under TEM. PF NPs exhibited spherical shapes with smooth surfaces (Figure 3A). The dynamic average particle size of PF NPs was measured to be 151.7 ± 5.87 nm, from 58.77 to 615.1 nm. It followed a unimodal distribution with the polydispersity of 0.249 ± 0.023, as displayed in Figure 3C. These results are reasonable and commonly accepted for nanoparticles in drug delivery systems (Zhang et al., 2018; Zhou et al., 2018).

PF NPs showed good dispersion and stability after they were resuspended in water; they maintained a stable and homogeneous status after the dispersion for 3 days at room temperature (Figure 3B).

CAC is an important parameter to characterize the structural stability and anti-dilution ability of polymeric nanoparticles. Pyrene selectively enters the hydrophobic cores of the nanoparticles, which change the polarity of the environment and cause the wavelength to shift from 334 to 336 nm (Figure 3D). The intensity ratio of the two peaks increased significantly, which indicated that nanoparticles began to form. By plotting the ratio of intensity to the negative logarithm of concentration, we determined the mutation point of the curve and calculated CAC of PLEL to be 3.06 × 10^–3^ g.L^–1^ (Figure 3E), which is 2∼5 orders of magnitude lower than those of common surfactants like sodium octane sulfate or potassium laurate. It is indicated that PLEL has a higher structural stability and stronger anti-dilution ability than some surfactants.

The entrapment efficiency (EE,%) and loading efficiency (LE,%) of PF-loaded nanoparticles were measured in order to demonstrate the drug releasing characteristics. The EE% and LE% of PF NPs formed under the present protocol were 72.3 ± 5.62% and 6.98 ± 0.53%, respectively, according to the calculation equations of (1) and (2). The standard curve of PF as a function of the absorbance at the wavelength of 246 nm followed the Eq. 3. The results are summarized in Table 2.

**TABLE 2:** Characterization of PF NPs.

	Particle size (nm)	Polydispersity	Zeta potential (mV)	LE (%)	EE (%)
PF NPs	151.7 ± 5.87	0.249 ± 0.023	−32.73 ± 3.43	6.98 ± 0.53	72.3 ± 5.62

*Data represented as mean ± SD, n = 3.*

Pranoprofen (PF) is a non-steroidal anti-inflammatory drug which is commonly used in clinic to relive anterior eye inflammation and postoperative pain. However, the poor water solubility of pure PF greatly reduced the treatment efficacy. After being encapsulated in amphiphilic PLEL as spherical nanoparticles, PF can be dispersed into water to become uniform suspension, which is believed to be helpful in reducing the drug clearance and improving the cellular uptake and bioavailability of PF in eye drop administration.

### *In vitro* Release Property of PF From Nanoparticles

**FIGURE 4:**
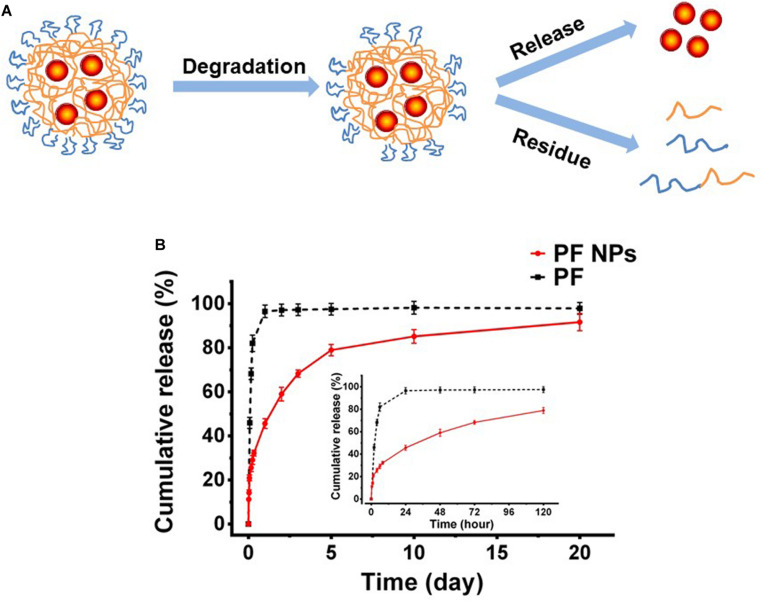
*In vitro* drug release. **(A)** Schematic diagram. **(B)** *In vitro* release tendency of PF from PF NPs with pure PF as the control (*n* = 3).

The mechanism of drugs releasing from nanoparticles involves diffusion or dissolution of the drug and degradation of the carrier. The initial diffusion of the drug came from the absorption on the nanoparticle surface or loosely mosaic in the polymeric matrix. Subsequently, the drug encapsulated in the carrier released slowly into the aqueous medium with water penetration and the fracturing of the hydrophilic PEG segments on the particles. Finally, the degradation of the bulk polymer took place and the drug released thoroughly from the carrier. This mechanism is schematically presented in Figure 4A. In this work, the *in vitro* releasing properties of PF from the nanoparticles were detected with the dialysis bag diffusion method. As shown in Figure 4B, it exhibited an initial burst—sustained—slow release profile of PF from PF NPs. PF released for ∼20% at the first 2 h and ∼60% in the next 5 days, followed by a slow release up to 20 days. On the contrary, the pure PF in the same dialysis bag was shown to release much faster; 82% of the drug permeated out of the dialysis bag in the first 6 h.

The initial burst release was due to the drug being adsorbed/loaded on the particle surface. After that, the drug released sustainedly as the polymer degraded till the amount leveled out. This kind of release profile would be beneficial for disease treatment in ophthalmology, because the initial burst release allowed the drug to reach an effective therapeutic concentration immediately after administration and the sustained release is conducive to maintaining the therapeutic effect in the eyes.

### *In vitro* Cytotoxicity Assessment of PLEL and PF NPs

**FIGURE 5:**
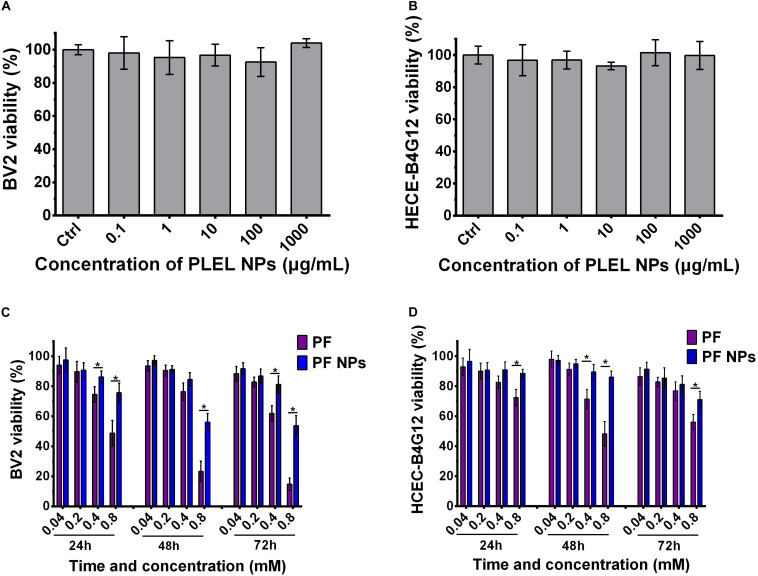
Cell viability of PLEL, PF, and PF NPs with various concentrations and testing times. **(A,B)** BV2 and HCEC-B4G12 cells cultured for 72 h (*n* = 5). **(C,D)** BV2 and HCEC-B4G12 cells were treated with pure PF and PF NPs from 24 to 72 h (*n* = 4). *Indicated a statistically significance (*p* < 0.05).

BV2 microglial cells and HCEC-B4G12 cells were used to detect the toxic effects of PLEL NPs and PF NPs on neurons and ocular cells, respectively. The results of CCK8 assays showed that the viability of cells treated by PLEL NPs with concentrations from 0.1 μg/mL to 1.0 mg/mL is almost similar to that of cells without any NPs (0 μg/mL) (*p* > 0.05), indicating that the material PLEL is not toxic to either cells (Figures 5A,B). The materials we synthesized are very biocompatible.

The cytotoxicity of PF NPs was tested with pure PF as the control. The viability of both types of cells decreased gradually with the increase of PF concentration at all culture time points, from 24 to 72 h. They displayed dose-dependent natures (Figures 5C,D). This result is consistent with the literature results reported by Li et al. (2015). For the BV2 cell, the viability had no significant difference for concentrations of 0.04 and 0.2 mM between groups of free PF or PF NPs treatment at all-time points. However, free PF at a concentration of 0.8 mM displayed a higher cytotoxicity compared with PF NPs at the same concentration (*p* < 0.05, Figure 5C). This tendency was also found in HCEC-B4G12 cells (*p* < 0.05, Figure 5D). It indicates that the drug PF itself is neurotoxic and toxic for ocular cells. In clinic, this drug was used to treat anterior eye inflammation and postoperative pain. However, the continuous use of the drug caused toxic keratitis, with symptoms of conjunctival congestion and eyelid redness, and, in severe cases, led to corneal epithelial damage or exfoliation. This is why we modify PF with PLEL encapsulation. With a slow release from the nanoparticles, the drug maintained anti-inflammatory efficacy but reduced the side effects on ocular cells.

### *In vivo* Tests

#### Ophthalmic Irritation

Draize Eye Test, one of the irritation test methods most commonly used in ophthalmic clinic, has often been adopted to evaluate eye impairment and is expressed as scores, with higher scores indicating more serious impairment (Draize et al., 1944). Corneal turbidity, iris congestion, conjunctival edema, and conjunctival secretion were scored at various times after drug administration. In our case, no edema or other injury were observed on the ocular surface after treatment with PS, PLEL NPs, PF, or PF NPs for 1, 4, 8, and 24 h in the single-dose irritation test. Therefore, the Draize test scores of eyes were 0.

**FIGURE 6:**
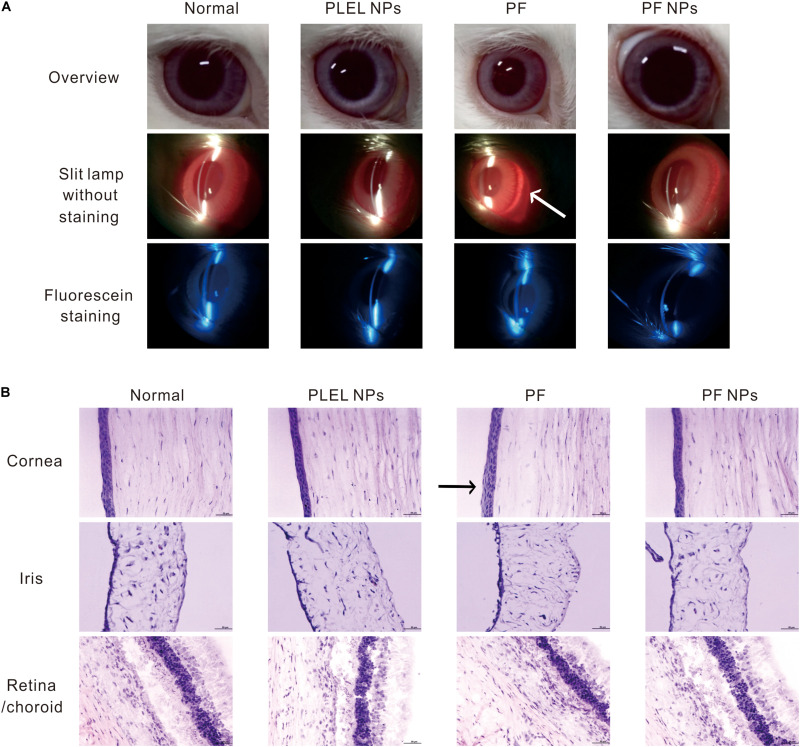
Ophthalmic irritation studies. **(A)** Ocular observation. Arrow indicates mild redness of the eye **(B)** Histology analysis (HE staining). The rabbits’ eyes were treated with physiological saline (PS), PLEL NPs, PF, and PF NPs formulations for seven consecutive days. Scale bar, 50 μm. Arrow indicates mild swelling in the corneal epithelium.

After eye drops for seven consecutive days three times daily, mild redness on the eyes was observed in the animal group of PF treatment (Figure 6A, arrow). The histological assay after eye tissues were collected and stained by H&E dyes and showed mild swelling in the corneal epithelium (Figure 6B, arrow), indicating long-term administration of the PF drug really irritated the animal eyes. However, no obvious corneal breaks were found under slit lamp observation after eyes were stained with sodium fluorescein, suggesting PF causes mild swelling of the cornea and iris, but no tissue broken. On the contrary, no edema or injury on the eyes treated with PLEL or PF NPs for seven consecutive days (three times daily) were observed under slit lamp. The same results for sodium fluorescein staining observation and HE staining for tissue sections were obtained (Figures 6A,B), indicating that PLEL NPs or PF NPs were not an irritant. Tissues of the cornea, iris, retina, and sclera were normal; no damage or abnormal cell infiltration was observed (Figure 6B). These results confirmed the good biocompatibility of the material PLEL, which was attributed to the good hydrophilicity of PEG and adapted to the humid environment of the ocular surface.

In summary, eye irritation tests have shown that a single dose of PF did not cause damage to the ocular tissue, but long-term use of the drug caused slight erythema of the top ocular. However, PF NPs did not irritate the eyes in both short-term and long-term usage. It can be safely used for ophthalmological treatment. This result is consistent with the cytotoxic test.

#### Anti-inflammation of PF NPs

**FIGURE 7:**
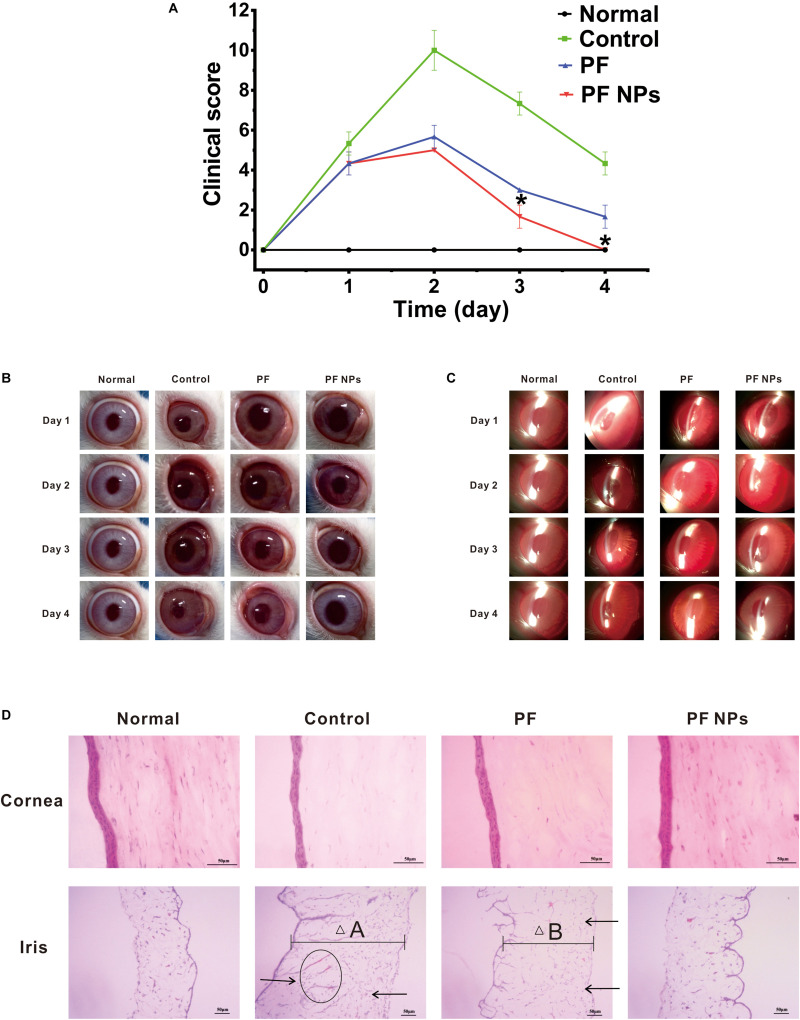
*In vivo* anti-inflammatory tests. **(A)** Score alteration after the infected eyes were treated with pure PF or PF NPs (1 mg/mL) for 4 days. The control eyes were treated with PS. *Indicates a statistically significance (*p* < 0.05). Ocular observation with eyes **(B)** and a silt lamp **(C)** were performed. **(D)** HE staining. Scale bar, 50 μm. Arrows indicate infiltration of inflammatory cells. The ellipse indicates neovascularization. “△A” and “△B” indicate tissue widths.

After LPS induction, inflammatory symptoms were observed in all eyes of animals, for example, swelling and congestion of the conjunctiva and iris, anterior chamber exudates, and abnormal tears on the ocular surface. After these infected eyes were treated with pure PF or PF NPs (1 mg/mL) from 1 to 4 days, we found that all eyes had the highest scores at day 2 according to the rules of Table 1 (Figure 7A). The eyes had various degrees of tissue swelling and congestion (Figure 7C). After eyes were treated with PS, in addition to swelling and congestion of different tissues, the cornea displays a mild haze and the abnormal secretion from the ocular surface moistened the eyelid at day 2, indicating that the inflammation was the most serious at that time (Figure 7B). Comparatively, the eyes treated with PF or PF NPs displayed less serious inflammation than those of the control, though there was no statistical difference between the eyes treated with PF or PF NPs. The inflammation score of the PF NPs-treated eyes was lower than that of the PF-treated eyes at both days 3 and 4. The score reached 0 at day 4, indicating complete recovery. But the iris of the PF-treated eyes still had moderate swelling and congestion, though the cornea was cured under a silt lamp observation (Figure 7C). Severe swelling or congestion of the iris and anterior chamber exudates were evident in the PS-treated eyes (without drug); the test score was still as high as 4.33 ± 0.58. These results showed that PF NPs had a better anti-inflammatory effect than PF did; the infected eyes can be cured by PF NPs more quickly than by PF. This could be attributed to the reduced irritation and sustained release of the drug after nanoparticle encapsulation.

After treatment for 4 days, the cornea and iris tissues were collected and stained by H&E dyes. The results showed that no inflammatory cell infiltration or neovascularization in cornea tissues of the eyes treated with PS, PF, or PF NPs were obvious (Figure 7D), which is consistent with the results of the top observation (Figures 7B,C). However, the remarkable swelling (Figure 7D, △A), inflammatory cell infiltration (Figure 7D, arrow), and neovascularization (Figure 7D, ellipse) in the iris parts were obvious in the PS-treated eyes. Particularly, the iris of PF-treated eyes showed mild swelling (Figure 7D, △B) and inflammatory cell infiltration (Figure 7D, arrow), while the iris of PF NPs-treated eyes showed a complete recovery. *In vivo* anti-inflammatory assessment demonstrated that PF NPs had stronger anti-inflammatory efficacy than PF alone; not only was the inflammatory seriousness reduced, but the tissue’s recovery progress was also accelerated. The elimination of immunological inflammation was also proven by other researchers, where polypeptide and rapamycin-loaded PLGA or stereocomplex micelle made from the enantiomeric 4-armed PEG/PLLA copolymer were fabricated toward effectively suppressing the progression of immune inflammation (Shengxian et al., 2018; Wang et al., 2018; Feng et al., 2019).

## Conclusion

In this work, PF NPs with an average particle size of 151.7 ± 5.87 nm and a smooth spherical surface were prepared using the method of emulsion solvent evaporation, aiming to improve the bioavailability of a drug that is topically delivered to the eye. As a nanoparticle carrier, the PLEL copolymer has no toxicity to ocular cells and little irritation to animal eyes, which shall be attributed to the biocompatible PLLA and hydrophilic PEG component in the copolymer molecules. After being encapsulated in amphiphilic PLEL spherical nanoparticles, the original hydrophobic PF can be dispersed uniformly into aqueous solution to form a stable suspension, which is believed to be helpful to reduce the drug clearance, improve the cellular uptake, and upgrade the bioavailability of PF in eye drop administration. The PF NPs showed to be less harmful to ocular cells and eyes than the pure PF drug. The PF NPs exhibited a sustained drug-releasing profile for up to 5 days, which is very useful against long-lasting anti-inflammatory with low administration frequency. The results of *in vivo* tests proved this assumption; the better anti-inflammatory effect of PF NPs on the infected eyes than that of pure PF was proved. Thus, we concluded that PF NPs is safer and the anti-inflammatory efficacy is more prolonged than in pure PF, which proves it to be a novel promising treatment in ophthalmology to relieve anterior eye inflammation and postoperative pain in the future. However, the effect of PEG content on the physicochemical properties of the polymer and the anti-inflammatory effect of PF NPs needs further study.

## Data Availability Statement

All datasets presented in this study are included in the article/supplementary material.

## Ethics Statement

The animal study was reviewed and approved by the Animal Ethics Committee of Ningbo University.

## Author Contributions

All authors listed have made a substantial, direct and intellectual contribution to the work, and approved it for publication.

## Conflict of Interest

The authors declare that the research was conducted in the absence of any commercial or financial relationships that could be construed as a potential conflict of interest.
